# Hypoxia Responses: How Different Cells and Tumors React to Oxygen Shortage

**DOI:** 10.1371/journal.pmed.0030105

**Published:** 2006-01-24

All cells require an adequate supply of oxygen to function. Oxygen is used in aerobic metabolism, which turns carbohydrates into the energy needed to power essential cellular processes such as protein synthesis. Cells, therefore, have to sense and respond to hypoxia—inadequate oxygen levels. In mammalian cells, a family of transcription factors (proteins that control when genes are transcribed) called hypoxia-inducible factors (HIFs) are particularly important in orchestrating cellular responses to hypoxia.

HIF-1—the first HIF to be identified—contains two subunits. HIF-1β is expressed all the time; HIF-1α is an oxygen-sensitive inducible subunit. When there is sufficient oxygen (normoxic conditions), enzymes called prolyl hydroxylases add hydroxyl groups to HIF-1α. This leads to the recognition of HIF-1α by von Hippel–Lindau (VHL) protein, which then adds a molecular tag to HIF-1α that targets it for destruction. When oxygen is limiting, the prolyl hydroxylases cannot work, so HIF-1α escapes destruction and forms an active transcription factor by dimerizing with HIF-1β. This induces the expression of numerous target genes that help cells to survive hypoxia. HIF-1α can also escape destruction in normoxic conditions if the VHL protein is defective or missing.

As well as their important role in normal cells, hypoxia responses are also important in many human diseases. In cancer, the lack of oxygen deep within the tumor or genetic changes can induce a strong hypoxia response that helps the tumor to grow faster, and reduces the efficacy of chemotherapy and radiotherapy. But, despite the importance of the hypoxia response in sickness and in health, it is known that a hypoxia response does not affect all tissues evenly. But little is known about how different cell types respond to hypoxia. DNA microarrays (“gene chips” that can synchronously interrogate thousands of genes for their expression levels) have proven to be very valuable tools to evaluate the similarity and differences between cell types. Jen-Tsan Chi, Zhen Wang, Patrick Brown, and an international team of researchers have remedied this by examining global changes in gene-expression programs in response to hypoxia in several different cell types.

The researchers grew four human cell types in normoxic, hypoxic, or anoxic conditions and determined which genes were expressed in each condition using DNA microarrays. They then used a mathematical approach to identify genes whose expression under hypoxic conditions varied according to cell type. They found that the expression of many more genes was induced in kidney epithelial cells than in breast epithelial cells; fewer genes still were induced in nonepithelial cells. To understand physiological and pathological responses to oxygen variability, it will be necessary to find out exactly what drives these tissue- and cell type–specific differences. For now, though, the researchers report that the vigorous transcriptional response to hypoxia in renal epithelial cells was, in part, due to high levels of HIF-1α expression in these cells. They also suggest that this strong hypoxia response may explain why patients with VHL disease, who have a mutated *VHL* gene, develop mainly kidney tumors.

The researchers next used gene-expression data from renal and breast epithelial cells to derive a gene-expression signature of the epithelial hypoxia response. Because tumor hypoxia is an important factor in human cancer progression, the researchers reasoned that such a signature might help to predict clinical outcomes in patients with various carcinomas (epithelial cell tumors). For kidney, breast, and ovarian cancers, the signature allowed the researchers to divide tumors into a high hypoxia response group and a low hypoxia response group. For breast and ovarian cancer, a high hypoxia response correlated with poor outcome.

Finally, Chi and colleagues investigated whether the gene-expression signature of hypoxia could indicate the likely outcome for patients any better than existing prognostic factors (for example, tumor size and grade). Their results indicated that the information provided by the hypoxia signature was more predictive than any of the clinical parameters currently in use, and independent of predictive information provided by a “wound” gene-expression signature constructed in a similar way. The researchers conclude that by integrating gene-expression characteristics of tumors with genetic changes and clinical data, it should be possible to build up a detailed biological profile of individual tumors that could be used both to predict their clinical course and to choose appropriate general or targeted therapies (for example, drugs designed to inhibit HIF activity) when these become available.

## 

**Figure:**
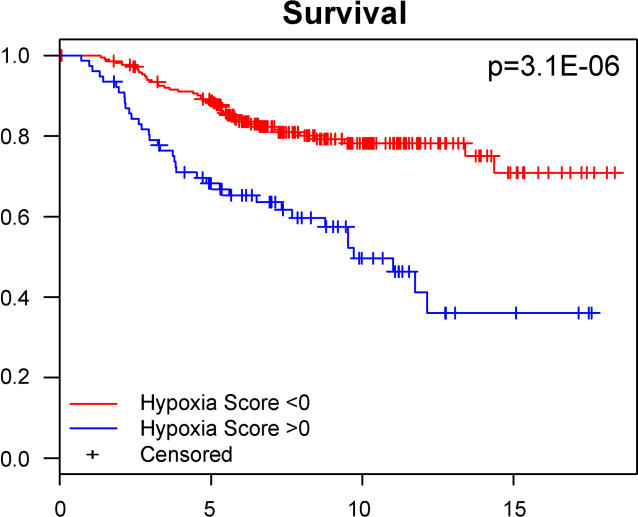
Hypoxia score is associated with patient survival

